# *Sgt1*, but not *Rar1*, is essential for the *RB*-mediated broad-spectrum resistance to potato late blight

**DOI:** 10.1186/1471-2229-8-8

**Published:** 2008-01-23

**Authors:** Pudota B Bhaskar, John A Raasch, Lara C Kramer, Pavel Neumann, Susan M Wielgus, Sandra Austin-Phillips, Jiming Jiang

**Affiliations:** 1 Department of Horticulture, University of Wisconsin-Madison, Madison, WI 53706, USA; 2 Biotechnology Center, University of Wisconsin-Madison, Madison, WI 53706, USA

## Abstract

**Background:**

Late blight is the most serious potato disease world-wide. The most effective and environmentally sound way for controlling late blight is to incorporate natural resistance into potato cultivars. Several late blight resistance genes have been cloned recently. However, there is almost no information available about the resistance pathways mediated by any of those genes.

**Results:**

We previously cloned a late blight resistance gene, *RB*, from a diploid wild potato species *Solanum bulbocastanum*. Transgenic potato lines containing a single *RB *gene showed a rate-limiting resistance against all known races of *Phytophthora infestans*, the late blight pathogen. To better understand the *RB*-mediated resistance we silenced the potato *Rar1 *and *Sgt1 *genes that have been implicated in mediating disease resistance responses against various plant pathogens and pests. The *Rar1 *and *Sgt1 *genes of a *RB*-containing potato clone were silenced using a RNA interference (RNAi)-based approach. All of the silenced potato plants displayed phenotypically normal growth. The late blight resistance of the *Rar1 *and *Sgt1 *silenced lines were evaluated by a traditional greenhouse inoculation method and quantified using a GFP-tagged *P. infestans *strain. The resistance of the *Rar1*-silenced plants was not affected. However, silencing of the *Sgt1 *gene abolished the *RB*-mediated resistance.

**Conclusion:**

Our study shows that silencing of the *Sgt1 *gene in potato does not result in lethality. However, the *Sgt1 *gene is essential for the *RB*-mediated late blight resistance. In contrast, the *Rar1 *gene is not required for *RB*-mediated resistance. These results provide additional evidence for the universal role of the *Sgt1 *gene in various *R *gene-mediated plant defense responses.

## Background

Potato late blight, a disease caused by the oomycete pathogen *Phytophthora infestans*, is one of the world's most devastating crop diseases. World-wide losses due to late blight exceed several billion dollars annually [1]. Most of the potato cultivars currently grown in the United States are highly susceptible to late blight and control of this disease relies almost exclusively on fungicide applications. The most effective and environmentally sound way for controlling late blight is to incorporate natural resistance into potato cultivars. The pedigrees of many potato cultivars currently used in different countries include late blight resistant germplasm derived from *Solanum demissum*, *Solanum andigena*, and other wild species. However, most of the resistance derived from these wild species is controlled by single dominant resistance genes (*R *genes). These *R *genes are only effective in preventing the development of late blight if the invading *P. infestans *race contains the corresponding avirulence genes. This *R *gene-mediated resistance is often short-lived and is rapidly overcome by new races of the late blight pathogen.

*Solanum bulbocastanum *(2n = 2x = 24) is a diploid species that has adapted in the same environment as the late blight pathogen. This wild species was characterized as possessing durable resistance against *P. infestans*, even under high disease pressure [2,3]. Two resistance genes, *RB *(*Rpi-blb1*) and *Rpi-blb2*, have been cloned from *S. bulbocastanum *[4-6]. Both genes confer broad-spectrum resistance against a wide range of known *P. infestans *races. Transgenic potato lines containing a single *RB *gene showed a high-level resistance in the Toluca Valley, Mexico, where the potato fields are naturally intensively infested with the most diversified *P. infestans *populations [7]. Most interestingly, transgenic *RB *plants did not show total immunity to late blight, but instead showed a marked delay in both onset of symptoms and development of lesions. Such rate-limiting resistance may put less selection pressure on the *P. infestans *populations and protect the durability of this resistance gene. The *RB *gene therefore provides an excellent model to study the mechanism of broad-spectrum and rate-limiting disease resistances. An understanding of the underlying mechanism of this type of resistance is important for developing strategies to breed durable and sustainable disease resistance.

Several genes have been implicated in the regulation of *R *gene function. Of these genes, *Rar1 *and *Sgt1 *are among the most extensively studied genes. The *Rar1 *(required for *Mla12 *resistance) gene was first identified for its essential role in the function of a subset of *Mla *genes that confer resistance to barley powdery mildew [8]. The RAR1 protein contains two highly similar but distinct cysteine- and histidine-rich (CHORD) Zn^2+^-binding domains and was proposed to play a role in stabilizing R proteins in a confirmation that is implicated in receiving pathogen signals [9]. The *Sgt1 *gene (suppressor of the G2 allele of *skp1*) is an essential gene with multiple functions in yeast. SGT1 protein was initially identified as a RAR1-interacting partner in a yeast two-hybrid screen [10]. SGT1 may play a role in R protein accumulation [11]. *Rar1 *and *Sgt1 *genes are required in various *R*-gene mediated resistance against viral, bacterial, oomycete or fungal pathogens [12]. However, none of the previously studied *R *genes showed a race-non-specific and rate-limiting resistance phenotype as the *RB *gene. In addition, the role the *Rar1 *and *Sgt1 *genes are not universal and these genes are not essential for resistance involving some *R *genes [12,13].

Besides the two broad-spectrum resistance genes *RB *and *Rpi-blb2*, several race-specific late blight resistance genes have also been cloned [14-16]. Numerous late blight resistance genes have recently been mapped in various potato species or populations [17-25]. However, there is almost no information available about the resistance pathways mediated by any of these genes. As an initial effort to understand the *RB*-mediated late blight resistance pathway, we silenced the *Rar1 *and *Sgt1 *genes using an RNAi-based approach in a potato line containing the *RB *gene. We demonstrated that SGT1, but not RAR1, is essential for the *RB*-mediated broad-spectrum resistance to potato late blight.

## Results

### Identification of the potato *Rar1* and *Sgt1* genes

A search of the Institute for Genomic Research (TIGR) potato database [26] using a sequence from the tobacco *Rar1 *gene (AF480487) identified a potato EST, TC121848 (1187 bp), which showed 96% sequence similarity to the tobacco and tomato *Rar1 *transcripts. Similarly, a search using a sequence from the tomato *Sgt1 *gene (TC85297) identified a potato EST, TC112395 (1461 bp), which showed 98% sequence similarity to the tobacco and tomato *Sgt1 *genes and 90% sequence similarity to the *Arabidopsis thaliana Sgt1b *gene. Since the full-length cDNAs for the *A. thaliana Rar1 *and *Sgt1b *genes are 901 and 1290 bp, respectively [27,28], the identified potato ESTs almost cover each of the complete potato genes. Southern blot hybridization was performed to determine the copy numbers of the *Rar1 *and *Sgt1 *genes in the potato genome. Genomic DNA was isolated from potato clone K41, which contains the *RB *gene introgressed from *S. bulbocastanum*, and was hybridized with the potato *Rar1 *and *Sgt1 *gene probes. The Southern hybridization results showed that the haploid potato genome contains only one copy of the *Rar1 *gene and two copies of the *Sgt1 *gene (data not shown), which agree with a similar conclusion reported by Pajerowska et al. (2005).

### RNAi-based silencing of the potato *Rar1* and *Sgt1* genes

**Figure 1:**
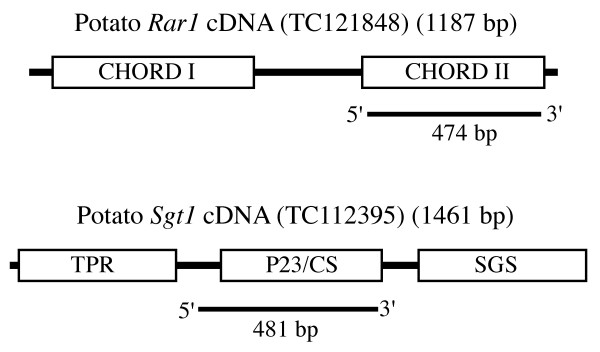
Schematic representation of the potato *Rar1 *and *Sgt1 *genes and regions used for RNAi construct development.

We developed RNAi constructs for the potato *Rar1 *and *Sgt1 *genes. The *Rar1 *silencing construct contained a 474-bp fragment covering the CHORD II domain. The *Sgt1 *construct contained a 481-bp fragment that targets the P23/CS domain (Figure 1). These constructs were used for *Agrobacterium*-mediated transformation of the potato line K41. We obtained 65 and 58 independent transgenic lines for the *Rar1 *and *Sgt1 *genes, respectively.

**Figure 2:**
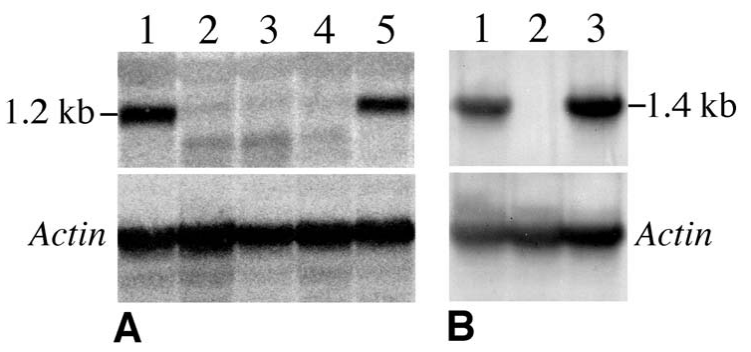
**Transcription analysis of *Rar1 *and *Sgt1 *silenced transgenic lines**. **(A) **Northern blot analysis of transcription of the *Rar1 *gene. Lane 1: a transgenic plant (clone 3007) containing the *Rar1*-RNAi construct but not silenced; Lane 2–4: three independent *Rar1*-silenced lines (clones 3128, 2998, 3028) showing no transcript; Lane 5: untransformed K41 control. **(B) **Northern blot analysis of transcription of the *Sgt1 *gene. Lane 1: a transgenic plant (clone 3061) containing the *Sgt1*-RNAi construct but not silenced; Lane 2: *Sgt1*-silenced line (clone 3095) showing no transcript; Lane 3: untransformed K41 control. Both blots were re-probed with an actin gene probe to ensure equal loading of mRNA in each lane.

The expression of the *Rar1 *and *Sgt1 *genes in the transgenic lines was analyzed by Northern blot hybridization using the *Rar1 *(1187 bp) and *Sgt1 *(1461 bp) genes as probes. A significant reduction of the *Rar1 *transcripts was observed in 47 of the 65 *Rar1*-RNAi transgenic lines analyzed (Table 1). *Sgt1 *transcript reduction was also observed in 35 of the 58 *Sgt1*-RNAi lines (Table 1). Three *Rar1*-RNAi lines and one *Sgt1*-RNAi line showed no detectable Northern hybridization signals after exposure for over one month using intensifying screens (Figure 2). Only these four lines were used in the late blight resistance evaluation. We observed no distinguishable morphological features associated with RNAi-induced silencing of the *Rar1 *and *Sgt1 *genes in these transgenic lines.

**Table 1:** RNAi Silencing efficiencies of the *Rar1 *and *Sgt1 *genes based on Northern blot hybridization

**Gene**	**Transgenic lines screened**	**Transgenic lines with non-detectable transcript**	**Transgenic lines with normal transcript level**	**Transgenic lines with partial transcript level**		**Silencing efficiency**
					
				**>50%**	**<50%**	
*Rar1*	65	32	18	7	8	72%
*Sgt1*	58	1	23	20	14	60%

>50% indicates that transcript loss was less than 50%

<50% indicates that transcript loss was more than 50%

### Late blight resistance evaluation of the *Rar1*- and *Sgt1*-silenced potato lines

**Figure 3:**
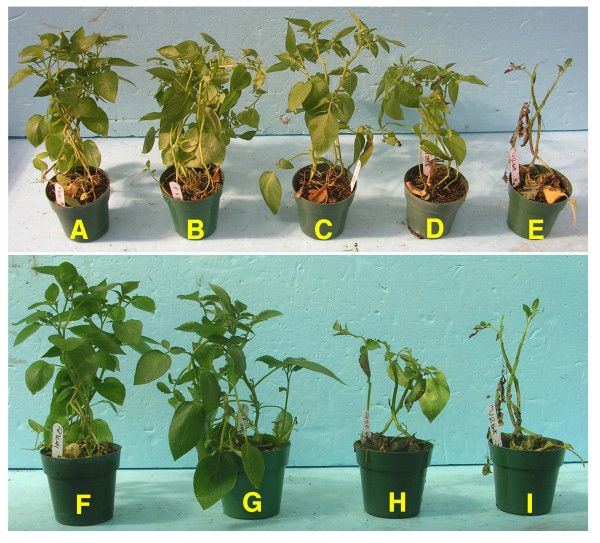
**Late blight resistance phenotype of *Rar1*- and *Sgt1*-silenced lines**. Controls and silenced plants were inoculated with *P. infestans *and photographs were taken seven days after inoculation. (**A, F**) Untransformed K41 control (*RB*+); (**B**) A transformed but not silenced *Rar1*-RNAi line (clone 3007); (**C, D**) Two independent *Rar1*-silenced lines (clones 3128, 2998); (**E, I**) Susceptible control Katahdin (*RB*-); (**G**) A transformed but not silenced *Sgt1*-RNAi line (clone 3061); (**H**) A *Sgt1*-silenced line (clone 3095).

**Figure 4:**
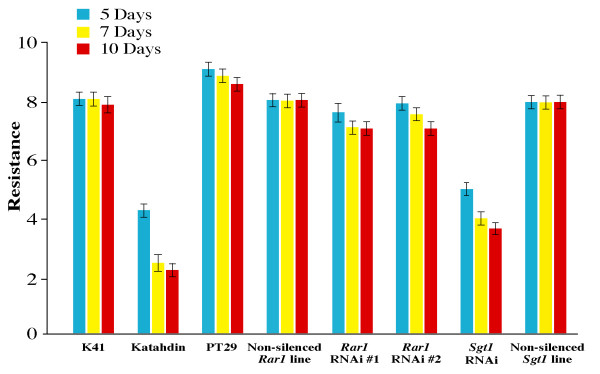
**Late blight resistance evaluation of *Rar1*- and *Sgt1*-silenced lines**. Resistance readings were taken 5, 7 and 10 days post inoculation (dpi). K41: untransformed control (*RB*+); Katahdin: susceptible control (*RB*-); PT29: a *S. bulbocastanum *clone as the resistant control (*RB*+); Non-silenced *Rar1 *line (*RB*+): a transgenic, but non-silenced *Rar1*-RNAi line (clone 3007); *Rar1 *RNAi line #1 (*Rar1 *silenced, *RB*+, clone 3128); *Rar1 *RNAi line #2 (*Rar1 *silenced, *RB*+, clone 2998); *Sgt1 *RNAi line (*Sgt1 *silenced, *RB*+, clone 3095); Non-silenced *Sgt1 *line (*RB*+): a non-silenced *Sgt1*-RNAi line (clone 3061). Error bars represent the standard deviation from the means.

We evaluated the late blight resistance of the *Rar1*- and *Sgt1*-silenced lines together with several controls that either contain or do not contain the *RB *gene, including transformed but not silenced *Rar1*-RNAi and *Sgt1*-RNAi lines (no transcript reduction was found in these RNAi lines). Triplicates of each line were used in each of two independent inoculation experiments. All variation within replicates and experiments were taken into account in determining resistance scores. The average score of the late blight infection on the *Rar1*-silenced plants was 7.3 (± 0.6) after 7 days post inoculation (dpi) and 6.9 (± 0.0) after 10 dpi, which represents ~13% and 18% foliage infection, respectively (Figures 3, 4). The untransformed K41 plants and the non-silenced *Rar1*-RNAi line showed an average score of 8.0 (± 0.0), representing less than 10% infection. All susceptible controls showed an average score of 2.1 (± 0.6), representing ~81% infection. ANOVA showed that there was a significant difference in mean resistance scores among the tested plants (P-val = 2.2e-16) after seven days. Fisher's Least Significant Difference (LSD) test as a comparison of resistance score means revealed no significant differences between the *Rar1*-silenced plants and other resistant controls but revealed significant differences with the susceptible control, Katahdin. These results show that the *RB*-mediated resistance was not affected in the *Rar1 *silenced lines.

The *Sgt1*-silenced plants showed an average late blight infection of 4.0 (± 0.0) after 7 dpi and 3.7 (± 0.7) after 10 dpi, representing ~70% and ~73% infection, respectively (Figures 3, 4). Significant differences among the mean resistance scores were observed among the tested plants starting from day seven using ANOVA with P-val = 2.2e-16. Fisher's LSD test revealed that the *Sgt1*-silenced plant had a significantly different resistance score compared to the resistant control (untransformed K41) and non-silenced *Sgt1 *plants. These results suggested that silencing of the *Sgt1 *gene compromised the *RB*-mediated late blight resistance.

**Figure 5:**
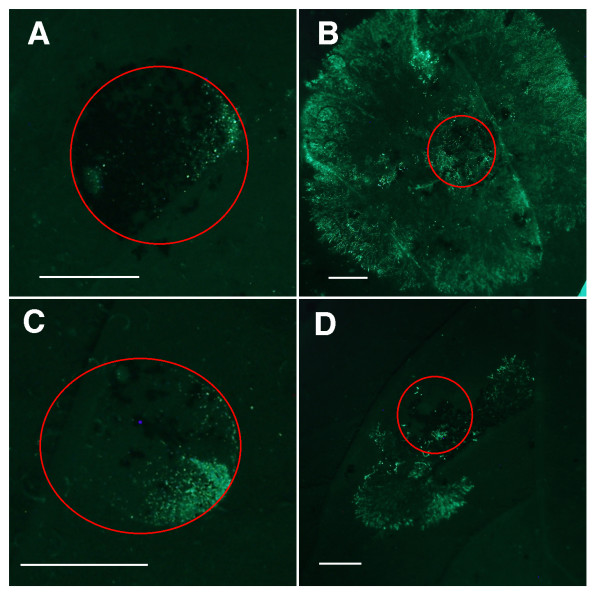
**Late blight resistance evaluation using a GFP-tagged *P. infestans *strain**. Leaves were inoculated on plants with GFP-tagged isolate 208m2 and were removed 6 days post inoculation and photographed on a dissecting microscope under a narrow-band GFP filter. **(A) **Untransformed K41 control. **(B) **Katahdin. **(C) **A silenced *Rar1*-RNAi line (clone 3128). **(D) **A *Sgt1*-silenced line (clone 3095). The red circles outline the original location of the 10 μl inoculation drops. Green fluorescence is only observed within the circles in A and C but spreads out of the original inoculation sites in B and D. Bars = 2.5 mm.

**Figure 6:**
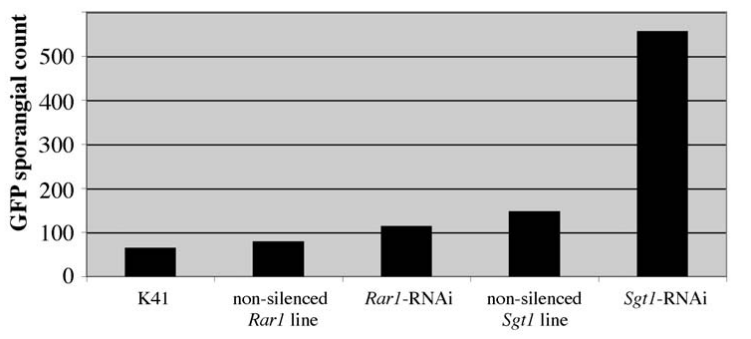
**Quantitative analysis of GFP *Phytophthora *lesions**. The average GFP sporangial counts (particle count) are shown from K41: untransformed control (*RB*+); Non-silenced *Rar1 *line (*RB*+): a transgenic, but non-silenced *Rar1*-RNAi line (clone 3007); A *Rar1*-RNAi line (clone 3128); Non-silenced *Sgt1 *line (*RB*+): a non-silenced *Sgt1*-RNAi line (clone 3061); A *Sgt1*-RNAi line (clone 3095). Using a one-way ANOVA, with unequal variance, and Fisher's LSD at an alpha of 0.01, the only group that has significantly higher sporangial growth is the *Sgt1*-silenced line.

The late blight resistance of the *Rar1*- and *Sgt1*-silenced lines were also evaluated using the GFP-tagged *P. infestans *isolate 208m2 [29]. The *Phytophthora *growth was quantified by counting the fluorescing sporangia within each area; more *P. infestans *growth is indicated by larger counts. One and three days after inoculation, no significant differences were observed between the sporangial counts on the silenced and non-silenced controls. Six days after inoculation, however, there were significant differences among the sporangial counts of the tested plants (Figure 5) based on a one-way ANOVA with unequal variance (F = 3.627, P-val = 0.03142). Fisher's LSD, using an alpha of 0.01, indicated that the *Sgt1*-silenced plant had significantly more *P. infestans *growth, as indicated by sporangial count, than the K41 control, the transgenic but not silenced *Rar1*-RNAi and *Sgt1*-RNAi lines and the *Rar1*-silenced plants (Figure 6). These results are consistent with those from the conventional greenhouse evaluations.

## Discussion

### The *Rar1* and *Sgt1 *genes in potato

*Rar1 *is a single copy gene in barley [10] and *A. thaliana *[28,30]. Our data from Southern blot hybridizations confirm the previous report that only one copy of *Rar1 *exists in the haploid potato genome [31]. Plant *rar1 *mutants show no visible growth defects [32], although silencing of the *Rar1 *homolog in *C. elegans *resulted in semi sterility and embryo lethality [8]. The *Rar1*-silenced potato plants did not show any visible growth phenotypes in our study.

SGT1 is an essential protein for proper kinetochore function in yeast and null mutations of the single copy *Sgt1 *gene are lethal [33]. Most plant species analyzed appear to contain two *Sgt1 *genes [11,31,34,35]. The *Sgt1a *and *Sgt1b *double mutant in *A. thaliana *is embryo lethal [11]. Similarly, virus-induced gene silencing (VIGS) of the *Sgt1 *genes (*NbSgt1.1 *and *NbSgt1.2*) in *N. benthamiana *caused a stunt phenotype [34]. In tomato, silencing of the *Sgt1-1 *gene, but not *Sgt1-2*, was lethal. This result was possibly caused by silencing of both the *Sgt1-1 *and *Sgt1-2 *genes using the VIGS construct designed for *Sgt1-1 *[35]. In contrast, silencing of the wheat and barley *SGT1 *genes by VIGS did not result in a growth phenotype [36,37]. However, the copy number and functional redundancy of the *SGT1 *genes in wheat and barley are not clear, this may be caused by either partial silencing of the gene and/or compensated by partially homologous and non-silenced *SGT *genes in these species.

We did not observe any abnormal growth phenotype in the *Sgt1 *silenced line. The complete silencing of the *Sgt1 *gene was confirmed by Northern blot hybridization (Figure 2B). The available sequences of the two potato *Sgt1 *genes (*StSgt1-1*: AY615272; *StSgt1-2*: AY615274) are 100% identical. The *Sgt1 *cDNA fragment used in our RNAi contruct also shares 100% homology to these sequences. These results suggest that both copies of the potato *Sgt1 *gene are likely silenced in the RNAi silencing line. Therefore, it appears that silencing both of the *Sgt1 *genes is not lethal in potato. This result will need to be confirmed by multiple completely silenced *Sgt1 *lines.

### Requirement of the RAR1 and SGT1 proteins in disease resistance

A role of the RAR1 protein in disease resistance signaling was first recognized in barley through *Mla12*-mediated resistance against powdery mildew [8]. The same role for RAR1 has since been identified in the resistance pathways conferred by several genes that belong to the NB-LRR family [12,36-38]. Interestingly, there were also reports that the *Rar1 *gene appears to not play a role in the resistance pathway. The RAR1-independent cases include the *Mla1*-mediated resistance against powdery mildew in barley [10,39], *Bs2/AvrBs2*-mediated resistance against bacterial spot disease in *N. benthamiana *[40], and *Mi-1*-mediated resistance against root-knot nematodes in tomato [35]. We demonstrated that silencing of the single *Rar1 *gene in potato does not affect the *RB*-mediated late blight resistance.

The previous RAR1-independent conclusions were based on gene silencing techniques by biolistic delivery of double stranded RNA constructs into single cells [10,39] or by VIGS [35,40]. These techniques may not result in complete suppression of a target gene and uniform silencing of the gene throughout the infected plants. False negative results have to be carefully sorted out because a low level of the transcripts resulting from incomplete silencing may be sufficient to facilitate the function of RAR1. The RNAi-silenced *Rar1 *potato lines showed no transcripts based on stringent Northern blot hybridizations (Figure 2), suggesting a near complete silencing of the *Rar1 *gene. These stable silenced lines allowed repeated resistance evaluations. Thus, our results from multiple RNAi silencing lines provide conclusive evidence that *RB*-mediated resistance is RAR1 independent.

The RAR1 protein has been proposed to be involved in forming or stabilizing the recognition complexes associated with R proteins or in assisting conformational changes of the recognition complexes [12]. Such functions may not be required in the complex assembly of a subset of R proteins. Bieri et al (2004) showed that in barley *rar1 *mutants that are compromised for MLA6 but not MLA1 resistance, the steady state level of both MLA isoforms is reduced. However, MLA6 accumulated to about a four-fold lower level than MLA1 in transgenic lines. Interestingly, *Mla1 *functions independently of *Rar1 *only where MLA1 abundance exceeds a threshold level [39]. A number of previous studies suggest that RAR1 may control the abundance of the NB-LRR type R proteins [39]. We propose that the RB protein level in the *Rar1 *silenced potato lines may be sufficient to trigger the resistance pathway.

Many previous studies confirmed the requirement of the SGT1 protein in resistance mediated by both NB-LRR and *Pto*-kinase type resistance genes, as well as in non-host resistance [34,35,37,40,41]. Several resistance genes in *A. thaliana *were SGT1b independent in the *sgt1b *mutant background [27,28,30]. However, SGT1a may complement the loss of SGT1b [11]. The MLA1-triggered resistance in barley, unlike other MLA variants, was largely unaffected when the *HvSgt1 *gene was silenced [10]. The transient silencing method used in this system may not have been complete, leaving some levels of HvSGT1 for MLA1 to function. Alternatively, MLA1 requires low levels of HvSGT1 to operate as compared to other MLA variants [10]. Bhattarai et al. (2007) recently showed that partial silencing of the *Sgt1-1 *gene in tomato resulted in attenuation of *Mi-1*-mediated potato aphid resistance, but the same plants still held the *Mi-1*-mediated root-knot nematode resistance. These results support the hypothesis that plant R proteins differ in the amounts of SGT1 needed to trigger effective resistance [11]. We demonstrate that silencing of *Sgt1 *clearly compromised the *RB*-mediated late blight resistance (Figures 3, 4, 5), indicating that the RNAi approach most likely silenced both copies of the *Sgt1 *gene and the *Sgt1 *gene plays the key role in the pathway. Our results provide further evidence for the universal role of the *Sgt1 *gene in various *R *gene-mediated plant defense responses.

## Conclusion

We developed RNAi-based silencing lines of genes *Rar1 *and *Sgt1 *in a potato line that contains the late blight resistance gene *RB*, which confers broad-spectrum resistance against all known races of *P. infestans*. Silencing of the *Rar1 *and *Sgt1 *genes in potato does not result in phenotypic changes. Late blight resistance evaluation of the *Rar1 *and *Sgt1 *silenced lines showed that the *Sgt1 *gene is essential for the *RB*-mediated late blight resistance. In contrast, silencing of the *Rar1 *gene does not abolish the *RB*-mediated resistance. These results provide additional evidence for the universal role of the *Sgt1 *gene in various *R *gene-mediated plant defense responses.

## Methods

### Plant materials

Potato line J101K6A6K41 (K41) is a tetraploid clone and was developed from the somatic hybrid J101 between potato and *S. bulbocastanum *(clone PT29) [42]. J101 was used as the female parent to backcross with Katahdin (BC1), Atlantic (BC2) and Katahdin (BC3). K41 is a late blight resistant BC3 clone. Presence of the *RB *gene in K41 was confirmed by polymerase chain reaction (PCR) using *RB*-specific primers [43] and this clonal line was used in transformation experiments designed to silence the *Rar1 *and *Sgt1 *genes. *S. bulbocastanum *(PT29) and potato cultivar 'Katahdin' were used as controls for late blight resistance evaluation.

### RNAi construct design and potato transformation

Total RNA was extracted from leaf tissue of K41 using the RNeasy Plant Mini Kit (Qiagen, Valencia, California) and treated with TURBO DNA-*free *(Ambion, Austin, Texas) to remove DNA contamination. First strand cDNA was synthesized using 1 μg of total RNA, oligo d(T) primer and superscript reverse transcriptase (Invitrogen, Carlsbad, California). The cDNA fragments used to silence *Rar1 *and *Sgt1 *were amplified by PCR. A 474-bp cDNA fragment from *Rar1*, corresponding to the TIGR potato EST TC121848 (nt 346 through 820), was amplified from K41 cDNA using Platinum *Taq *DNA polymerase (Invitrogen). The primers used for amplification included 5' CACC CAA CAC CAT CTG CTA CCA AAA A 3'(forward) and 5' GAC ACT GGG TCA GCG TTG TG 3'(reverse). A 481-bp cDNA fragment from *Sgt1*, corresponding to the TIGR potato EST TC112395 (nt 359 through 840 bp) (this EST is recently split into TC133190 and TC159283) was amplified from K41 cDNA using primers 5' CACC GGC CTG TAT GAA GCT TGA AGA A 3'(forward) and 5' TCT GCA TTT TGC AGG TGT TAT C 3' (reverse). The *Rar1 *and *Sgt1 *amplicons were successively purified using QIAquick PCR purification kit (Qiagen), gel verified and then cloned into pENTR/D-TOPO vector using the pENTR Directional TOPO Cloning kit (Invitrogen). The *Rar1 *and *Sgt1 *DNA fragments were then transferred into the pHellsGate8 vector using the LR Clonase recombination method according to Helliwell et al. (2002) [44]. The sequence-verified pHellsgate8-*Rar1 *and pHellsgate8-*Sgt1 *constructs were then transformed into K41 using standard *Agrobacterium*-mediated transformation protocols [45].

### Gel-blot hybridizations

Southern blot hybridization was performed according to Stupar et al. (2002) [46]. Genomic DNA was isolated from leaf tissues of K41 and digested with restriction enzymes. The DNA blots were probed with a 1-kb genomic DNA fragment of the *Rar1 *gene and a 1.1-kb genomic DNA fragment of the *Sgt1 *gene to evaluate the copy numbers of these two genes in the potato genome. RNA blots were prepared using 10–15 μg of total RNA isolated from leaf tissue using the TRIzol protocol (Invitrogen). To verify the transcription of the *Rar1 *and *Sgt1 *genes in the RNAi lines, RNA blots were hybridized with the complete cDNA fragments of *Rar1 *(TC121848) and *Sgt1 *(TC112395) amplified from K41 cDNAs. Hybridizations were performed as described previously [47]. Intensifying screens were used to detect the Northern blot hybridization signals and the blots were exposed for at least one month to reveal if any transcripts were detectable in the *Rar1*- and *Sgt1*-RNAi lines.

### Late blight resistance evaluation

*Rar1 *and *Sgt1 *silenced plants along with several controls were evaluated for late blight resistance in environmentally controlled greenhouses at the University of Wisconsin-Madison Biotron facility. Controls include the susceptible potato cultivar Katahdin (without *RB *gene), *S. bulbocastanum *(PT29), non-transformed K41 lines and K41 lines that were transformed with the construct containing either *Rar1 *or *Sgt1*, but not silenced. The inoculation and resistance evaluation were performed as described previously [43]. Briefly, the selected lines were grown in triplicates and were randomly placed in a mist chamber eight hours prior to inoculation. The mist chamber held a 24-hour relative humidity of 100%, an 8-hour light period, a daytime temperature between 17–19°C and a nighttime temperature at 13–15°C. The plants were inoculated with 76,000–80,000 sporangia/ml of sporangial suspensions of *P. infestans *isolate US930287 (US-8 genotype, A-2 mating type). Measurements of the foliage blight were interpreted and scored according to the Malcolmson scale [48]. The scale was based on percent of foliage infected and scores were as follows: 9 – no visible infection; 8 – <10% infection; 7 – 11–25%; 6 – 26–40%; 5 – 41–60%; 4 – 61–70%; 3 – 71–80%; 2 – 81–90%; 1 – >90%; 0 – 100% infection. Blight scores were recorded 5, 7 and 10 days after inoculation. An average score for the resistance was determined using the three replicates of each clone in each inoculation experiment.

### Resistance evaluation using a GFP-tagged *P. infestans* strain

A quantitative method was employed using a strain of *P. infestans*, which contains GFP, to better quantify pathogen growth. All silenced lines, as well as the transgenic but not silenced *Rar1*-RNAi and *Sgt1*-RNAi lines, were inoculated by the GFP-tagged *P. infestans *isolate 208m2 provided by Dr. Felix Mauch (University of Friberg, Switzerland) [29]. The final average sporangial concentration was 64,259 sporangia/mL, using a hemocytometer. The sporangial suspension was placed at 15°C for three hours before inoculation to release the zoospores. One 10 μl drop of the suspension was placed on both sides of the midvein, on the bottom of the leaf, in approximately the same location. Two leaves were sampled from each plant 24 and 72 hours after inoculation and four leaves were sampled 144 hours (six days) after inoculation. Each leaf was examined for the presence of actively growing *Phytophthora *using an Olympus SZX12 dissecting scope. Each area was photographed with an Olympus DP70 digital camera using the 41020 Chroma narrow-band GFP filter with an excitation of 425/75 nm and emission of 500/50 nm.

The spreading *Phytophthora *on each leaf surface were quantified by first converting the GFP images to black and white. The color conversion highlights the GFP *Phytophthora *sporangia and mycelium growth within each area. The area was then outlined and analyzed using the "analyze particle" tool in the ImageJ software [49]. This tool scans the image selection until it finds the edge of an object, corresponding to fluorescing sporangia or mycelium on the leaf surface. The tool outlines each object measures it and fills it in. It counts this small area as one particle and resumes scanning until it reaches another particle, where it repeats the process until the end of the image selection. The data report details the total number of counted particles, representing the total number of fluorescing sporangia on the leaf surface. As the *Phytophthora *grows, more sporangia and mycelium are present, which translates into a higher particle count. We measure the pathogen growth directly, not just the lesion, or area of dead tissue present on the leaf surface, which may not correspond to the actual pathogen spread. This experiment was designed as a Complete Randomized Design (CRD) with a total of 56 observations for days one and three and 50 observations for day six. Data quality tests and assessments were performed, such as residual and qq-plots. A one-way ANOVA with unequal variance was performed to compare the particle counts for each of the silenced lines with the empty vector controls and Fisher's LSD tested each group individually.

## Authors' contributions

PBB and JJ conceived the project. PBB developed RNAi constructs. JAR and SA developed transgenic lines. PBB and PN characterized transgenic lines. PBB, LCK, and SMW conducted disease resistance evaluation. PBB and JJ drafted the manuscript. All authors read and approved the final manuscript.
